# Supplementary material for: Natural Variation of Fatty Acid Desaturase Gene Affects Linolenic Acid Content and Starch Pasting Viscosity in Rice Grains
Source: Int J Mol Sci. 2022 Oct 10;23(19):12055. doi: 10.3390/ijms231912055 (PMC9570344; doi:10.3390/ijms231912055)
Supplement: Supplementary file 1 [file ijms-23-12055-s001.zip › Supplementary Figures S1-S3.pdf]

Natural Variation of Fatty Acid Desaturase Gene Affects Linolenic Acid Content and Starch Pasting Viscosity in Rice Grains

Liting Zhang [1,2], Yu Xia [1,2], Yage Dong [1,3], Tianyi Xie [1,2], Wenqiang Sun [1,3], and Sibin Yu [1,2,4,*]

[1] National Key Laboratory of Crop Genetic Improvement, Huazhong Agricultural University, Wuhan 430070, China

[2] College of Plant Science and Technology, Huazhong Agricultural University, Wuhan 430070, China

[3] College of Life Science and Technology, Huazhong Agricultural University, Wuhan 430070, China

[4] Hubei Hongshan Laboratory, Wuhan 430070, China

* Correspondence: ysb@mail.hzau.edu.cn

Supplementary Figures S1-S3

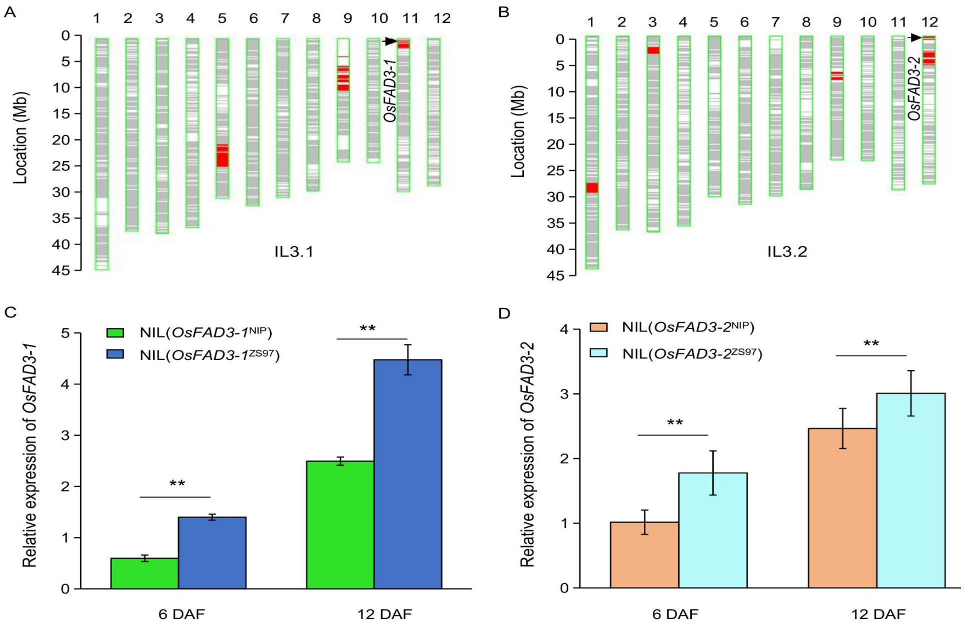

Figure S1. Graphic genotype of IL3.1 and IL3.2 and expression analyses of *OsFAD3-1* and *OsFAD3-2* in the spikelets of the near-isogenic lines (NIL). (A, B) Graphic genotype of IL3.1 and IL3.2. (C, D) Expression analyses of *OsFAD3-1*and *OsFAD3-2* in the spikelets of 6 DAF and 12 DAF (day after flowering). The *Ubiquitin* as an internal reference. Values are means ± SD (n = 3 replications). Significance is indicated by *t*-test. **, P< 0.01.

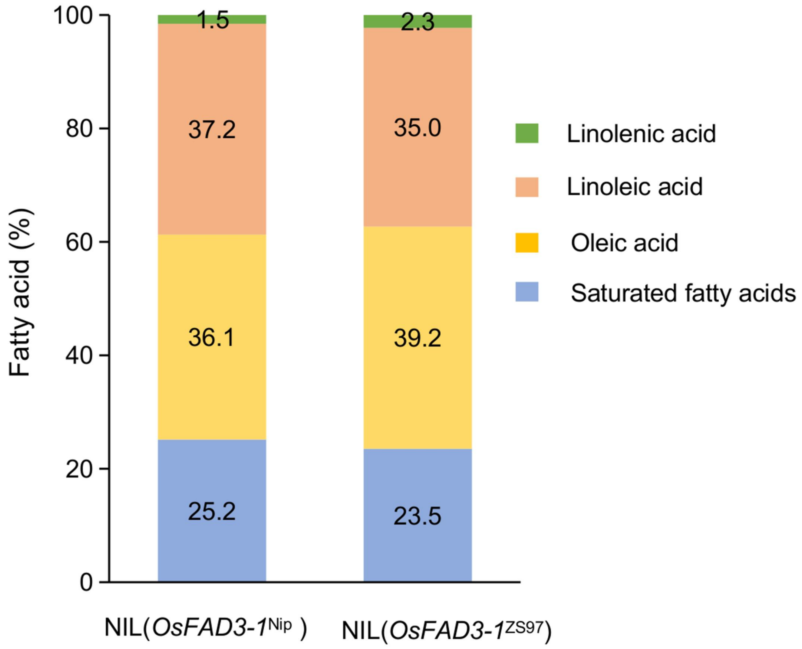

Figure S2. Relative contents of fatty acid compositions in the embryos of NIL (*OsFAD3-1^{Nip}*) and NIL (*OsFAD3-1^{ZS97}*).

A

```
Nip : GCTAGTTTTACGTATGGGGCTGGTTAGCTGTAATGCGCTACATATATGTTATTAGTATTG:-418
ZS97: GCTAGTTTTACGTATGGGGCTGGTTAGCTGTAATGCGCTACATATATGTTATTAGTATTG:-415

Nip : TAGGCAAATGAGTATGGAGTAGTAACAAATAGTAGTATGGATTTTGTTGAATAGAGATGG:-358
ZS97: TAGGCAAATGAGTATGGAGTAGTAAGAAATAGTAGTATGGATTTTGTTGAATAGAGATGG:-355

Nip : AGGGAGGACGGTTTAATTGAGGAGGCAAGAGAATCCAAAAAAGCATAAGCGTGTGGTGTG:-298
ZS97: AGGGAGGACGGTTTAATTGAGGAGGCAAGAGAATCCAAAAAAGCATAAGCGTGTGGTGTG:-295

Nip : GTGATGAAATGGGAAGGAAGGAGCAGAGGAGGCCTATAAATAGGGACATAGCAAAGCAAA:-238
ZS97: GTGATGAAATGGGAAGGAAGGAGCAGAGGAGGCCTATAAATAGGGACATAGCAAAGCAAA:-235

Nip : GGGGGCAGGCCGCACACAGCACAGGAGGAGGAGCTTGCTTTCCTTCCTTCCTTCCTTCCT:-178
ZS97: GGGGGCAGGCCGCACACAACACAGGAGGAGGAGCTTGGTTT--------CCTTCCTTCCT:-183

Nip : GGGTTGGGTTTGGGTTTGCGTTGCGGCCCATCCATCGCATTCGCGTTCGCAGGCACAAAC:-118
ZS97: GGGTTGGGTTTGGGTTTGCGTTGCGGCCCATCCATCGCATTCGCGTTCGTAGGCACAAAC:-123

Nip : CAAACACCTCCCCTCAAATTTCTCTTTCTTGTCTCTTCTCCCTTTCCGCCTCGCCTCGCT:-58
ZS97: CAAACACCTCCCCTTAAATTTCTCTTTCTTGTCTCTTCTCCCTTTCCGCCTCGCCTCGCC:-63

Nip : GCCAAA-----GCTTCACAAATCACAAATCGGAATCAGATCCACCACGACACGGCGGCGG:-3
ZS97: TCGCTGCCTGCGCTTCACAAATCACAAATCGGAATCAGATCCACCACGACACGGCGGCGG:-3

Nip : CA:-1
ZS97: CA:-1
```

B

```
NIP : MAASATQEADCKASEDARLFFDAAKPPPFRIGDVRAAIPAHCWRKTPLRSLSYVARDLLI:60
ZS97: MAASATQEADCKASEDARLFFDAAKPPPFRIGDVRAAIPAHCWRKTPLRSLSYVARDLLI:60

NIP : VAALFAAAASSIDLAWAWA-WPLYWAAQGTMFWALFVLGHDCGHGSFSDSAMLNNVVGHL:120
ZS97: VAALFAAAATRIDVSVAWAAWPLYWAAQGTMFWALFVLGHDCGHGSFSDSAMLNNVVGHL:120

NIP : LHSFILVPYHGWRISHRTHHQNHGHIERDESWHPITEKLYWQLETRTKKLRFTLPFTLLA:180
ZS97: LHSFILVPYHGWRISHRTHHQNHGHIERDESWHPITEKLYWQLETRTKKLRFTLPFTLLA:180

NIP : FPVYLWYRSPGKTGSHFLPSSDLFSPKEKSDVIVSTTCWCIMISLLVALACVFGPVPVLM:240
ZS97: FPVYLWYRSPGKTGSHFLPSSDLFSPKEKSDVIVSTTCWCIMISLLVALACVFGPVPVLM:240

NIP : LYGVPYLVFVMWLDLVTYLHHHGHNDLPWYRGEEWSYLRGGLTTVDRDYGWINNIHHDIG:300
ZS97: LYGVPYLVFVMWLDLVTYLHHHGHNDLPWYRGEEWSYLRGGLTTVDRDYGWINNIHHDIG:300

NIP : THVIHHLFPQIPHYHLVEATKAARPVLGRYYREPEKSGPLPLHLFGVLLRSLRVDHFVSD:360
ZS97: THVIHHLFPQIPHYHLVEATKAARPVLGRYYREPEKSGPLPLHLFGVLLRSLRVDHFVSD:360

NIP : VGDVVYYQTDHSLNGTDWAEDAKHK:385
ZS97: VGDVVYYQTDHSLNGTDWAEDAKHK:385
```

Figure S3. Comparison of sequence variation of *OsFAD3-1* in the varieties Nipponbare (NIP) and Zhenshan 97 (ZS97). (A) Promoter sequences, showing the 8-bp and 5-bp indels in red font letters; (B) Amino acid sequences of *OsFAD3-1*; The amino acid changes are indicated by red letters.
